# The Quail Game Farming Sector in Spain

**DOI:** 10.3390/ani12223118

**Published:** 2022-11-11

**Authors:** Francisco P. Caravaca, Tania Camacho-Pinto, Pedro González-Redondo

**Affiliations:** Departamento de Agronomía, Escuela Técnica Superior de Ingeniería Agronómica, Universidad de Sevilla, 41013 Sevilla, Spain

**Keywords:** advertising, alternative farming, characterisation, commercialisation, *Coturnix* spp., game farms, poultry, quail

## Abstract

**Simple Summary:**

In recent decades, populations of several game species in the wild have decreased, which has given rise to the creation of game farms that produce birds to be released in, and to restock, hunting preserves. Some game farming sectors are well known and developed, but in Spain, quail game farms have received little attention in terms of research. To address this gap in the literature, we characterised the Spanish quail game farm sector by administering a survey to farmers. We found that complete-cycle farms, of very different sizes, coexist with a majority of farms that do not have breeding flock, and which carry out only the finishing phase of quail raising. These farms mainly produce quails with good flying abilities that are primarily intended to be released for intensive hunting and shooting practices, rather than for restocking hunting preserves. Quails are sold almost all year round, and the farms also offer complementary services to clients, such as transporting birds to their destination, or organising bird releases at the customer’s hunting preserve. Quail game farms use various channels to promote themselves in a competitive market. These game farms originated in Spain five decades ago, which has led to the creation of a consolidated sector.

**Abstract:**

Quail (*Coturnix* genus) game farms were characterised in Spain using variables related to their age, geographical distribution, farmed species, structure, size, and commercialisation, using a survey conducted on 21 farms in 2018. It was found that 38.1% of the sample represented complete-cycle farms, and 61.9%, which have no breeding flocks, undertook only the finishing phase of quail raising. The average size of the breeding flock, with regard to complete-cycle farms, was 1096 males and 3735 females, with a female/male ratio of 3.6, and 75% of these farms carried out self-replacement of breeding quails. The most farmed species was European quail (*Coturnix coturnix*), followed by Japanese quail (*Coturnix japonica*), and hybrids of both species. In addition to quail, all farms produced other gamebird species such as pheasant (*Phasianus colchicus*) and red-legged partridge (*Alectoris rufa*). The rearing phase, which began when the chicks were one-day-old, lasted 35.3 days on average (range: 25–49 days), with an average stocking density of 47.2 birds/m^2^ in the brooder house. The finishing phase, which was carried out in flying pens at a stocking density of 9.5 birds/m^2^, ended when the quails’ average age was 60.5 days. All farms sold quails for release in hunting reserves (EUR 1.54 per bird) and for dog training (EUR 1.65 per bird) for almost 10 months of the year; only 62% sold quails for shooting after being thrown with an adapted clay-pigeon shooting machine (EUR 1.49 per bird). Transporting quails to their destination (95.2% of the farms) and releasing the birds in the client’s hunting preserve (52.4% of the farms) were services offered by the farms, among others. The main advertising and promotional strategies undertaken by the quail game farms to gain a share of the market included maintaining a business website (85.7% of the farms) and attending game and agricultural fairs (47.6% of the farms). In conclusion, this alternative poultry sector has been consolidated in Spain, five decades since the establishment of the first game farms, and it satisfies a relevant part of the demand for quail hunting.

## 1. Introduction

Quails of the *Coturnix* genus (mainly, *Coturnix coturnix* and *Coturnix japonica*) are raised in game farms for hunting purposes, among other uses. In several European countries such as Italy [1], Greece [2], the Republic of Serbia, Montenegro, Romania, Spain [1,3,4], and others, these farm-reared quails are released in hunting preserves in order to ensure or increase the number of hunting bags [3,5,6]. Put-and-take shooting, training dogs to retrieve birds, or shooting quails that are thrown with an adapted clay-pigeon shooting machine, are the most common uses for game farm-reared quails. The European, or common quail (*Coturnix coturnix* Linnaeus, 1758), that is autochthonous to Spain, has been identified as a bird species with an unfavourable conservation status [7]. Since the 1970s, in Spain the number of annually hunted quails has decreased by around 30% [5]; indeed, around 1,200,000 quails were hunted during 2019 [8]. This situation generated a demand for quails, to be used for hunting purposes, that has been satisfied by game farms.

The main species of quails that are being farmed for the hunting market are the common or European quail [9,10] and the domestic Japanese quail (*Coturnix japonica,* Temminck and Schlegel, 1849). Hybrids of both species are also being marketed more frequently, which are usually obtained by crossing female Japanese quails with common quail males, and successive generations are backcrossed with common species males. The latter two types of quail are more easily farmed than the European quail because of their better reproductive performance [5,11,12,13,14]. The European quail species is most appreciated by hunters due to its wild behaviour, whereas the Japanese and hybrid quails are less accepted by the hunting community due to their tame character and poorer flying ability. In addition, Japanese or hybrid quails releases for restocking purposes are banned in several European countries because they are not native species [2,11,12,15]. Due to their wild character, purebred European quails are difficult to farm and, in addition, they have a shorter reproductive season and a fewer number of eggs laid per female, compared with domestic Japanese quails or the hybrids [10,16].

The game quail farming model [9,11,14,17] is similar to that of other gamebirds, such as partridges and pheasants [14,18]. These gamebird species are subject to a similar reproductive management regime; breeding quails are housed indoors in battery cages, and two raising phases are completed before the quails are sold as hunting birds. The chicks are first reared in brooder houses on the litter floor (rearing phase), and they are subsequently raised in large open-air flight pens to exercise their flying ability until they are sold (finishing phase). There are also farms in which the rearing phase is carried out by housing the chicks in battery cages, until they are moved to the flight pens. The specialisation of the sector has led to two main types of quail game farms: complete-cycle farms and finishing farms (with no breeding flock) in which, starting at around 40 days of age, quail chicks are raised in flight pens. Moreover, there are few farms without breeding flocks where one-day-old chicks are raised until their sale. In complete-cycle farms, the female reproductive cycle begins between 42–45 days of age, and it lasts until the birds are between 5–12 months, depending on the species reared [9,11,14,17]. Reproductive performance increases if artificial photoperiod supplementation is applied to the breeding birds [9,14,17]. Artificial incubation is used to hatch the chicks [11,14].

Game quail farming and hunting-based activities are complementary resources for the rural sector [4,11] with a relevant socio-economic impact [5]; however, to date, this sector has not been sufficiently studied. Therefore, the aim of the present study is to characterise the game quail farming sector in Spain by using variables that are related to farm structure and management, as well as marketing and advertising strategies. This will provide valuable knowledge on this alternative poultry farming system and its diversity.

## 2. Materials and Methods

### 2.1. Study Area and Sample Selection

The study was conducted in Spain by administering a survey to quail game farmers. It followed the same methodology that has been previously applied to other Spanish game farming sectors in order to characterise them [19,20,21]. Several sources (public registers, enterprise databases, press advertisements, web searches, and personal contacts) were used to find farms, and they were subsequently invited to take part in the survey. All participating farmers were contacted and invited to join the study voluntarily and with informed consent. The sample used consisted of 21 farms, located in 9 regions (Table 1), whose stratified distribution was representative of the current regional distribution of the quail game farms registered in Spain [22]. The surveyed farms represented about 8% of the target population of farms that were being studied. The study only included private farms that commercialise their products; farms that produced birds to self-supply their hunting preserves were excluded.

### 2.2. Data Collection and Variables Studied

**Figure 1:**
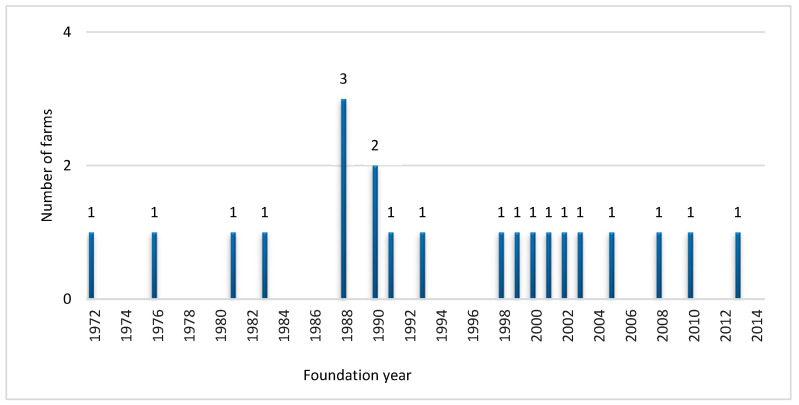
Number of quail game farms according to the year of establishment (n = 21).

The information was obtained using a survey carried out in May and June 2018, wherein farmers were directly interviewed. The structured questionnaire included qualitative and quantitative variables (Figure 1 and Tables 1–9) arranged into the following groups: (i) geographic location; (ii) year of establishment; (iii) farm size and breeding flock structure; iv) birds produced per year; (v) reproductive management; (vi) breeding flock replacement practices; (vii) facilities and management during the rearing and finishing phases; (viii) farmers’ knowledge of the types of quail they raise from a genetic standpoint; (ix) game species other than quail raised on the farm; (x) animal products supplied by the farm; (xi) additional services offered by the farm; (xii) market’s geographical area; and (xiii) farm advertising channels.

All variables were selected on the basis of a review concerning previous knowledge of the quail game farm subsector [4,9,11,14,23].

### 2.3. Statistical Analysis

The mean, standard error (SE), minimum and maximum were calculated for (i) the number of females, number of males, and the female-to-male ratio variables in the breeding flock; (ii) the number of housing facilities; (iii) stocking densities; (iv) bird ages during the different breeding and raising stages; and (v) mortalities that occurred during the two raising phases. Regarding the other variables of the survey, the number and percentage of farms showing those particular attributes were calculated. Chi-square tests were performed on contingency tables to analyse the differences between complete-cycle farms and farms that carry out only finishing phase, regarding the variables that were related to: (i) game species other than quail produced by the farm, (ii) additional services, other than the sale of birds, offered by the farm, and (iii) advertising and promotional channels chosen by the farm. Pearson correlations were performed in order to analyse the relationship between stocking density, on the one hand, and the length of artificial lighting, on the other, and mortality during the rearing phase. Moreover, Pearson correlations were also performed to assess the relationship between stocking density and mortality during the finishing phase of quail raising. Statistical analyses were performed using SPSS v.15.0 software [24].

## 3. Results

### 3.1. Year of Establishment

Figure 1 shows the years in which the surveyed farms were established. The oldest farm was founded in 1972, 1988 was the year in which more farms (n = 3) began to rear quail, and the 1990s and 2000s were the decades in which most of the quail farms were established (six farms per decade).

### 3.2. Farm Size, Breeding Flock Structure, and Birds Produced

Only 38.1% (n = 8) of the 21 surveyed farms were complete-cycle farms. The remaining 61.9% (n = 13) of farms raised quails by only carrying out the finishing phase of quail-rearing in flight pens. Most of the finishing farms (n = 12) reared quails by buying chicks aged 25 to 60 days old (42 ± 3.8 days; mean ± SE) from other farms, and the remaining one finishing farm bought one-day-old chicks and undertook also the starting or rearing phase in brooder houses. It is relevant to point out that the biggest quail farm in Spain, located in the region of Catalonia, supplied quail chicks for most of the finishing farms (n = 6; 46.2%). Some of these farmers preferred not to raise their own chicks, starting from own breeding flocks, because they consider the reproduction, incubation, and starting phases of the quail-rearing process to be unprofitable, due to the low market prices of one-day-old chicks.

When considering only complete-cycle farms, the average breeding flock was composed of 1096 males and 3735 females; the average female/male ratio was 3.6 (Table 2).

The average farm production was 76,824 ± 30,015.2 quails per year, though there was a great difference between the farm that produced the lowest number of quails (5000 quails) and the farm that produced the most quails, which sold around 580,000 birds per year. The number of quails produced by the latter complete-cycle farm is more than one third of the total number of quails produced by all of the surveyed farms (1,460,000 quails). Therefore, it clearly creates an unbalanced view of the sector because, when this big complete-cycle farm was excluded from the analysis, the average production decreased to 58,200 ± 18,953.6 birds/year for the complete-cycle farms (n = 5) or 45,308 ± 14,565.2 birds/year in the case of the finishing farms (n = 13).

### 3.3. Reproductive Management and Breeding Quail Replacement Practices

The surveys showed that reproductive life of breeding quails begins at an average age of 49 ± 2.3 days old, and it ranges between 39 (minimum) and 60 days old (maximum). The end of the reproductive life of quails occurs at an average age of 10.4 ± 1.07 months old, and it ranges between the ages of 5 and 12 months old. Most of the complete-cycle farms (n = 6; 75%) reported that the reproductive life of quails ends at 12 months of age, whereas the remaining two complete-cycle farms (25%) stated that the reproductive life of quails ends at 5 and 6 months, respectively; these farms were the smallest in terms of size and they produced purebred European quails. Two farms (25%) that raised Japanese quails or the hybrid quails reported that the reproductive life of quails ends at 12 months of age. However, four farms (50%) reported that the reproductive life of their quails ends at 12 months of age, and they raised the European quail species.

Most of the complete-cycle farms (75%) carried out self-replacement of their breeding flock, whereas the rest of the farms, in addition to the system of self-replacement, also bought breeding quails from other farms.

All complete-cycle farms housed breeding quails in battery cages that were kept indoors. In order to improve laying performance, all complete-cycle farms, except one, used artificial lighting during the laying period; however, each farm adopted different lighting schedules and durations.

### 3.4. Facilities and Management during the Rearing and Finishing Phases

The rearing phase began when the chicks were one-day-old, and it lasted between 25 and 49 days (35.3 days on average; Table 3). During this phase, the quails were housed at an average density of 47.2 birds/m^2^, with a minimum of 20 and maximum of 80 quails/m^2^. The average number of brooder houses was 12.4, and it ranged between one and 38, depending on farm size. The mortality during this phase averaged at 4.4%, and it ranged between 2 and 8%. No correlation was found between stocking density and mortality during the rearing phase in the brooder house (r = 0.434; *p* = 0.283; n = 8). The use of artificial lighting programs during this phase in complete-cycle farms (n = 8) ranged between no artificial light implementation (12.5%) and 24 h of artificial light (62.5%); other farmers supplied 16 h (12.5%) or 12 h (12.5%) of artificial light per day during this period. No correlation was observed between the length of the artificial lighting period and mortality rate during the rearing phase in the brooder house (r = 0.456; *p* = 0.257; n = 8).

The finishing phase is implemented by housing the quails in open-air flight pens, and it lasted, on average, 21.1 days, and it varied between 7 and 30 days (Table 3). The average stocking density in the flight pens was 9.5 quails/m^2^, ranging between one and 30 birds/m^2^. This phase started, on average, when the birds were 39.4 days old (range: 25–60 days), and it finished when the quails were sold, when they were an average age of 60.5 days old (range: 45–85 days). The average number of flight pens, where quails were housed during this phase, was 5.2, and it ranged between one and 38, depending on farm size. The mortality during the finishing phase was 1.9%, on average, and it ranged between 1 and 9%. No correlation was found between the stocking density and mortality during the finishing phase in the flight pens (r = 0.076; *p* = 0.744; n = 21).

### 3.5. Farmers’ Knowledge of the Genetic Type of Quail That They Raise

Table 4 shows the number and percentage of farms that raised a certain species of quail. Most of the farmers (76.2%) stated to produce the European quail, whereas the rest of them admitted that they produced the Japanese quail (14.3%), or a hybrid of the European and Japanese quail species (9.5%).

### 3.6. Game Species Other Than Quail That Were Raised on the Farms

All of the surveyed farms produced game species other than quails. Regardless of whether they were complete-cycle farms or carried out only the finishing phase of quail-raising (*p* > 0.05), three quarters of the farms also produced red-legged partridges (*Alectoris rufa*) or pheasants (*Phasianus colchicus*) in addition to quails, and half of them produced all three species concurrently (Table 5).

### 3.7. Products and Additional Services Offered by the Farms

Table 6 shows a descriptive analysis of the variables related to the products offered by quail game farms, such as selling periods and sale prices, depending on the birds uses (quails for release in hunting preserves, for dog training, or for shooting after being thrown with an adapted clay-pigeon shooting machine). All farms sold quails for their release on hunting preserves and for dog training, for 10.2 months of the year, on average, with a price of EUR 1.54 and EUR 1.65 per quail, respectively. The number of farms that sold quails for shooting after being thrown with a clay-pigeon machine was lower (62%); this product had a similar average price to the quails for release in hunting preserves, and it was available for 11.2 months of the year. Minimum (EUR 0.76 for quails for release on hunting preserves) or maximum prices per quail (EUR 2.50 for quails that are used in dog training) depended on the number of purchased animals. Hatching eggs, or one-day-old chicks, were products that were only offered by one of the surveyed farms (the largest one).

Table 7 shows the services offered by the farms in addition to the sale of birds. Most of the quail game farms offered their customers the service of transporting quails to their desired destination (95.2%). Around one third of the farms (38.1%) had their own hunting preserve, where they offered hunting and shooting activities for clients using the quails reared by the farm; only half of the farms (52.4%) were able to organise bird releases at the client’s hunting preserve, by request. No differences (*p* > 0.05) between farm types (complete-cycle or finishing farms) were found in terms of the services offered.

### 3.8. Market Geographical Area and Farm Advertising Channels

Table 8 shows the geographical area impacted by the quail game farms from a market perspective. Most quail game farms were marketing their products within the country (Spain), and the areas surrounding the farms (local or regional) were the most common areas that were reached. Only 23.8% of the farms sold their products throughout the whole Spanish territory, being all of them complete-cycle farms. Regardless of whether they were complete-cycle farms or farms that carry out only the finishing phase, 33.3% of the farms exported game quails, mainly to France and Portugal. The farms that exported quails were marginally more likely (*p* < 0.1) to be complete-cycle farm than finishing farm.

Nearly 90% of the quail game farms had their own website (Table 9); websites represented the main way in which the farms promoted themselves, in comparison to other promotional channels. Almost half of the farms promoted themselves by attending some of the numerous livestock and game fairs that are celebrated all over Spain; this was the second-most common means of promotion. One third of the farms advertised their products and services in hunting magazines that are currently being published in Spain. The Internet (14.3%), general press (4.8%), and livestock related press (4.8%) advertisements were channels that were used less often to promote the sale of quails, and the additional services offered by quail game farms. No differences were found (*p* > 0.05) between complete-cycle farms and farms carrying out only the finishing phase, in terms of promotional and advertising activities.

## 4. Discussion

There is little scientific [4,10,16,17,25,26,27] and informative literature [9,11,14,23] regarding quail game farming; indeed, to the best of our knowledge, this research provides the first systematic characterisation of Spanish quail commercial game farms, as a result of directly interviewing farmers, and through the study of variables related to their structure, management, marketing, and advertising channels.

Although quail farming for hunting purposes began earlier in other countries, such as the USA and Italy [28], some of the surveyed quail game farms were first established in Spain during the early 1970s (Figure 1), in accordance with that reported by Pérez y Pérez [23]. Quail game farms emerged contemporaneously with the establishment of the first red-legged partridge game farms [4,18,19], and in Spain, production both of these species was considered to be pioneer in terms of game farming. In fact, the commercial game farming of quails began in Spain before the game farming of pheasants (which started in 1980 [20]) and wild rabbits (which started in 1975 and became increasingly popular from 1990 [21]). Most of the surveyed quail farms were established between 1988 and 2003 (Figure 1), when other main game species were being farmed (red-legged partridge, pheasant, and wild rabbit) and consolidated as an important part of a new, alternative farm sector in Spain [18,19]. This might have been a consequence of the technological progress that was being made in terms of breeding and incubation techniques [29], the production of specific feed for gamebirds [30], as well as the promotion and extension on game species farming by companies, game organisations, and technicians, which was being carried out during this period [18,31]. The drastic decline in wild quail populations [4,7], and the growing demand due to the high number of hunters (more than 1,200,000 hunting licenses [32]) that took place in the 1990s, also contributed to the spreading of quail game farms across Spain.

Alternative poultry farming was considered to be an available option for farmers with few economic resources since it enables them to reuse abandoned lands and old facilities [33] to develop an interesting complementary activity. This may explain why all surveyed quail game farms raised more than one game species (Table 5). This has also been seen in characterisation studies of red-legged partridge [19] and pheasant [20] game farms in Spain, of which 27% and 76% of them, respectively, produce more than one game species. Only 19% of Spanish wild rabbit game farms, however, produce other game species [21]. Many of these farms raised a combination of quails, red-legged partridges, or pheasants because their husbandry uses common technologies and facilities with respect to artificial egg incubation, chick rearing in brooder houses, and bird rearing in flight pens during the finishing phase of bird raising [9,14,18]. Therefore, quail game farming is not an exclusive farming activity, and it is usually associated with farming other game species in order to cover most of the possible activities and demands that are related to game market.

Regarding the average size of complete-cycle farms, the data obtained in this study present a broad range of breeding flocks, ranging from one hundred up to 15,000 females (Table 2). This diversity is due to the fact that there are farms that have reached a profitable business size, in addition to smaller farms that engage in other, complementary farming activities, as has been previously seen in other game farming subsectors [19]. In fact, quails are often produced on red-legged partridge game farms as a secondary product (Empresa de Gestión Medioambiental, 2007, cited in González-Redondo and Sánchez-Martínez [21]). The breeding female/male ratio (3.6 females per male) recorded in the present study (Table 2) is in accordance with the ratio that was recommended for quail game farming in Riesco [14] (2–4 females per male), and it is slightly higher than the ratio described by Caballero de la Calle and Peña [11] (2–3 females per male) and Dalmau [9] (3 females per male). With regard to the number of birds produced per year, the total annual production of surveyed farms, excluding the largest farm, was 880,000 birds, whereas the largest farm reported a total annual production of 580,000 quails. Therefore, the total production of surveyed farms showed an evident polarisation in terms of quail production as just one of the farms concentrated around 40% of the commercialised game quails in Spain.

The beginning and end of the breeding quails’ reproductive life reported in this study for the quail game farms are in accordance with the reviewed literature on general quail farming [9,34]. However, several authors agree that the female quails’ reproductive performance decreases between 5–6 months [9] and 10–12 months [34], and that the use of artificial lighting contributes to a better reproductive performance [9,14,17], which is why almost all of the complete-cycle farms in our study use supplementary artificial lighting. The period of time in which eggs are produced is significantly related to genetic purity. The European quail species has a shorter laying season (around four months) [10], particularly when kept outdoors [17], due to the reproductive seasonality typical of a wild animal [23], whereas the Japanese species has an egg laying period that lasts up to 12 months [9,34,35]. However, four of the surveyed farms that were reported as rearing the European quail species bred quails which had a laying period of 12 months, which casts doubt on the genetic purity of the breeders.

Breeding quail self-replacement, which is a strategy followed by all quail farms (and that is the only replacement practice in three quarters of the surveyed farms), is also the same self-replacement strategy used in most game farms raising red-legged partridge [19], pheasant [20], and wild rabbit [21] in Spain. Although continuously maintaining the self-replacement strategy across many generations can lead to inbreeding problems [36], most farms follow this replacement strategy because the wild nature of game species made more difficult their reproduction in captivity, compared with domestic livestock species; thus, game farmers choose the offspring of their best quails as future breeders, in order to obtain better reproductive results.

Data obtained for the rearing phase length in the brooder house (Table 3) are in accordance with the reviewed literature. Indeed, Dalmau [9] and Caballero de la Calle and Peña [11] recommend an indoor rearing period of 25–30 and 40–45 days, respectively, for game quails. The bird density data obtained during the rearing phase (Table 3) are also within the range of recommendations in the literature: 30–40 quails/m^2^ [11] to 50 quails/m^2^ [9]. National law regarding the management of poultry farming [37] does not set recommendations or limitations on the stocking density of quails when they are being raised. The mortality during the rearing phase (Table 3) was lower than the 7.5–10% reported by Blanco [34] for Japanese quail and by Caballero de la Calle and Peña [11] for game quail. Although it is well-known that the stocking density and lighting parameters are factors that affect the welfare and health of quails [38,39], the absence of an association between both parameters with the relatively low mortality rate observed during the rearing phase, could be an indicator of the good management of birds in quail game farms.

Most farms decide the beginning of finishing phase (Table 3) in accordance with climatic conditions; therefore, moving birds to the flight pens may be delayed if necessary, particularly in rainy weather [9,11,14]. In this survey, the period of time in which quails remain in the flight pens (Table 3) was in the lower level of the interval referred to in the literature, which ranges between 15 days [14] and 30–45 days [11], with longer periods being advisable to improve their ability to adapt to field conditions in the wild. In this study, the quail density recorded during the finishing phase, in the flight pens (Table 3), was below the range that was recommended in the literature, at 20 birds/m^2^, thus permitting to improve birds’ physical condition to achieve a vigorous and fast fly and to minimise feather pecking, which devalues birds [9]. The mortality in this period (Table 3) was mainly caused by bad climatic conditions, and registered very low values. In fact, no association was found between stocking density and mortality in the flight pens.

As releasing Japanese or hybrid quails for restocking purposes is forbidden in most European countries because they are not native species [2,11,15], many of the farms affirm to produce the common or European species (Table 4), although without a genetic guarantee. For management and economic reasons, most farmers prefer breeding females displaying a longer laying season and greater level of productivity, which this implies the use of hybrid or domesticated quails rather than the common species. Consequently, for this variable, the veracity of the data arising from the survey should be questioned because many of the interviewed farmers that claimed they produced the European species cannot guarantee the purity of the species. Releasing quails other than the common species (hybrid or Japanese) for restocking purposes may cause biodiversity problems (alien genes introgression) and a decrease in the population of the native species. In order to reduce the likelihood of either circumstance, a reliable genetic control of farm-reared game quails should be established in order to certify the origin of the birds when they are released for restocking [40]. In fact, several Spanish regions have legislation in force or in project regarding this matter [41].

The main product offered by all game quail farms is a bird with excellent flying abilities, to be released and shot, mainly in private hunting preserves, throughout the year (Table 6). In addition, the intensive hunting preserves where clients hire an organized hunt with releases of quails, red-legged partridges and pheasants [4], often jointly, are becoming more and more frequent. All of the surveyed farms also sell quails to train hunting dogs in order to track and retrieve gamebirds that have been shot (Table 6) [9]. A somewhat lower proportion of farms (62%) offer quails for shooting via machine-throwing (Table 6), which is a hunting modality in which the birds are launched with a clay-pigeon shooting machine that has been adapted to launch live quails [9]. This is a controversial practice that is being banned in an increasing number of regional districts in Spain, and the Spanish Government is planning to enforce a nationwide ban [42] because it threatens animal welfare; therefore, the commercialisation of this type of bird is restricted to those provinces where shooting birds that are thrown from machines is not yet forbidden, though it will probably be fully banned by 2023.

According to statistical data from MITECO [8], in 2018, the total economic value of the 1.04 million quails hunted in Spain amounted to EUR 1.5 million. It is important to highlight that all of the surveyed farms reported a total annual production of 1.46 million birds. Although many of the hunted quails were wild, comparing the number of produced quails with hunted quails, it can be assumed that the surveyed farms were able to cover a large part of the total national demand, and they were able to generate enough in terms of surplus for foreign markets. The average sale price of the quails obtained in this work coincided with the value reported in the statistical data from MITECO [8] (EUR 1.5 per quail). The objective of these game farms is to sell birds for most of the year in order to satisfy the demand for the quails’ different uses, and thus, to guarantee a regular income.

Compared with red-legged partridge farms [18], game quail production has a low seasonal pattern; therefore, two thirds of the interviewed farmers offered their products throughout the year (Table 6). This is made possible due to the high likelihood that the breeding flocks used in most of these farms were either of a hybrid or domestic genetic type [11,14], because purebred European quails display a marked reproductive seasonality [10]. In fact, in Spain there are many intensive hunting preserves where hunting is authorised all year round [43], thus generating a continuous demand for quails.

Services supplied to customers by the quail game farms, as evidenced in the present study (Table 7), coincided with those reported in previous characterisation studies of game farms in Spain [19,20,21]. The proportion of quail game farms that transport birds to their destination, at the request of the customer (Table 7), was at a similar level to the proportion of red-legged partridge (85.7% [19]), pheasant (88% [20]), and wild rabbit game farms (85.7% [21]) that offer the same service. The high proportion of game farms offering customers the possibility of delivering purchased animals to their destination is explained by the fact that transporting game species is not easy to carry out due to their wild nature and tendency to become stressed [44]. Moreover, this also occurs because the requirements for transporting animals, according to European Union regulations, are complex [45]. Organising quails releases in the client’s preserve (Table 7), upon request, is a service offered by fewer quail game farms than red-legged partridge (84.1% [19]) and wild rabbit (44% [21]) farms that advise customers on how to release the animals; however, the number of quail game farms that offered this service was slightly higher than pheasant game farms (44% [20]). These differences are probably due to the different techniques used when releasing these species [9,14]. The proportion of quail game farms that have their own hunting preserves, or are associated with a hunting preserve that satisfies the customers’ demands for intensive shoots that use the quails produced by the farm (Table 7), is similar to that of red-legged partridge farms (36.5% [19]), lesser than that of pheasant farms (52% [20]), and greater than that of wild rabbit farms (9.5% [21]).

Compared with other game farming sectors in Spain (73.0% of red-legged partridge [19], 64.0% of pheasant [20], and 76.2% of wild rabbit [21] farms), the surveyed quail farms have a lower percentage in terms of commercialisation on a nationwide scale (Table 8), being the quail market more restricted to the nearby local and regional areas. This is possibly due to the fact that many quail game farms are located in geographical environments where the demand for birds for release is highest (Table 1). Regarding trade exports (Table 8), the values obtained for quail game farms are similar to those obtained for red-legged partridge [19] and wild rabbit [21] farms in previous studies reporting that one third of those farms were also able to export their game products mainly to bordering countries such as Portugal and France. However, only 12% of pheasant game farms had ever exported birds [20]. The export of quails to Portugal and France only is possibly due to the limitations on travel times established by European Union legislation in order to ensure the welfare of livestock during transportation [45], which makes it inoperative to ship them further afield.

The creation and maintenance of a business website appears to be the main promotion channel used by quail game farms in Spain (Table 9), probably due to its relatively low cost [46] compared with other classical promotion activities. The increase in the use of this promotional channel has been significant compared with previous research results in other game farming sectors in Spain (44.4% in red-legged partridge farms [19], 64.0% in pheasant farms [20], and 38.1% in wild rabbit [21] farms). In an environment in which communications and commerce through the Internet are widespread tools in business, the hunting sector has a long way to go in order to take advantage of the use of said websites as a marketing tool. Websites facilitate the transmission of a corporate image differentiated from competitors, and they also transmit information on the characteristics of the product offered to those in the hunting sector. In this sense, well-prepared and well-managed websites allow game farms to gain a competitive advantage in a market that sells animals for release in hunting preserves, a market that faces increasing levels of competition [47]. Promotion via attending livestock and game fairs was another of the preferred ways to promote the quail game farms and sell their products (Table 9); the popularity of this promotional activity has also increased when compared with other previous studies in related game sectors (20.6% in red-legged partridge farms [19], 44.0% in pheasant farms [20], and 19.1% in wild rabbit [21] farms). On the contrary, advertising their products and services in the hunting press (Table 9) has lost much relevance in quail game farms compared with what was described a few years ago for red-legged partridge (66.7% [19]), pheasant (56% [20]), and wild rabbit (42.9% [21]) farms. All other promotional and advertising channels were irrelevant (Table 9).

## 5. Conclusions

This research shows that the production of quails in game farms in Spain is a half-century old, and it has become a consolidated sector. Quail game farms vary markedly in terms of size, with one of them producing more than a third of all quails released in Spain, and they have evolved into two differentiated typologies. Some of the farms are complete-cycle farms, which involves breeding flocks, the artificial incubation of eggs, chick rearing in brooder houses, and finally, they are brought to flying pens; however, the majority of farms only carry out the finishing phase of chick raising in the flight pens. The main product is a bird that can be released in hunting preserves for intensive shooting, rather than for restocking. Moreover, it is marketed practically all year round, all over Spain, and several farms export quails to Portugal and France. Many of the farms offer services in addition to selling birds, which mainly involves the transportation of quails to the hunting preserves. The farms use various promotional and advertising channels, mainly their websites, which allows them to compete in a sector that has already matured.

## Figures and Tables

**Table 1:** Regional distribution of the Spanish quail game farms, according to the census (in 2018), and the sample of farms surveyed in this study.

Region	Census ^1^		Sample	
	n	%	n	%
Andalucía	12	4.5	9	42.9
Islas Baleares	3	1.1	-	-
Canarias	2	0.8	-	-
Cantabria	1	0.4	-	-
Castilla-La Mancha	9	3.4	1	4.8
Castilla y León	12	4.5	1	4.8
Cataluña	9	3.4	3	14.3
Comunidad Foral de Navarra	1	0.4	-	-
Comunidad Valenciana	5	1.9	2	9.5
Extremadura	196	74.2	1	4.8
Galicia	6	2.3	-	-
La Rioja	2	0.8	2	9.5
Comunidad de Madrid	2	0.8	1	4.8
Región de Murcia	1	0.4	-	-
País Vasco	3	1.1	1	4.8
Total	264	100	21	100

^1^ Dirección General de Producciones y Mercados Agrarios. Ministerio de Agricultura, Alimentación y Medio Ambiente [22].

**Table 2:** Average size of breeding flock and the female/male ratio in complete-cycle quail game farms.

Variable	Minimum	Maximum	Mean ± SE
Breeding males (n)	48	5000	1096 ± 668.6
Breeding females (n)	144	15,000	3735 ± 2012.8
Female/male ratio	3	5	3.6 ± 0.30

SE: Standard error.

**Table 3:** Variables related to facilities and management during the rearing and finishing phases of quail game farming (n = 21).

Variable	Minimum	Maximum	Mean ± SE
Rearing phase (n = 8)			
Rearing phase length (days)	25	49	35.3 ± 3.41
Brooder houses (n)	1	38	12.4 ± 4.38
Density in brooder house (birds/m^2^)	20	80	47.2 ± 7.44
Mortality during rearing phase (%)	2	8	4.4 ± 0.71
Finishing phase (n = 21)			
Starting age in flight pens (days)	25	60	39.4 ± 2.72
Sale age (days)	45	85	60.5 ± 2.32
Time in flight pens (days)	7	30	21.1 ± 1.70
Flight pens (n)	1	38	5.2 ± 1.70
Density in flight pens (birds/m^2^)	1	30	9.5 ± 1.69
Mortality in flight pens (%)	1	9	1.9 ± 0.40

SE: Standard error.

**Table 4:** Frequency and percentage of game farms that raised certain genetic types of quail.

Species	n	%
European quail (*Coturnix coturnix*)	16	76.2
Japanese quail (*Coturnix japonica*)	3	14.3
Hybrid	2	9.5
Total	21	100.0

**Table 5:** Frequency (n, and % between parentheses) of farms that produced game species other than quail (n = 21).

Species	Finishing Farms	Complete-Cycle Farms	Total	*p*-Value
Red-legged partridge (*Alectoris rufa*)	10 (76.9)	6 (75.0)	16 (76.2)	1.000
Pheasant (*Phasianus colchicus*)	10 (76.9)	5 (62.5)	15 (71.4)	0.631
Both species	7 (53.8)	4 (50.0)	11 (52.4)	1.000

**Table 6:** Sale price (EUR, Euro), and the number of months per year that the birds were sold, for the different products offered by quail game farms (n = 21).

Products Offered	Quails for Release (n = 21)		Quails for Dog Training (n = 21)		Quails for Machine-Throwing Shooting (n = 13)	
	Selling Period (months)	Sale Price (EUR)	Selling Period (months)	Sale Price (EUR)	Selling Period (months)	Sale Price (EUR)
Minimum	2	0.76	2	0.76	8	1.15
Maximum	12	2.30	12	2.50	12	2.30
Mean ± SE	10.2 ± 0.60	1.54 ± 0.08	10.2 ± 0.60	1.65 ± 0.09	11.2 ± 0.41	1.49 ± 0.08

SE: Standard error.

**Table 7:** Frequency (n, and % between parentheses) of additional services offered by quail game farms depending on farm type (n = 21).

Services Offered	Finishing Farms	Complete-Cycle Farms	Total	*p*-Value
Transport service	12 (92.3)	8 (100.0)	20 (95.2)	1.000
Organises birds releases at the client’s hunting preserve	8 (61.5)	3 (37.5)	11 (52.4)	0.387
Organises birds releases at the farm-owned hunting preserve	5 (38.5)	3 (37.5)	8 (38.1)	1.000

**Table 8:** Frequency (n, and % between parentheses) of market geographical areas reached by quail game farms, depending on farm type (n = 21).

Market Area Reached	Finishing Farms	Complete-Cycle Farms	Total	*p*-Value
Spain	0 (0.0)	5 (62.5)	5 (23.8)	0.003
Exports quails to other countries	2 (15.4)	5 (62.5)	7 (33.3)	0.056

**Table 9:** Frequency (n, and % between parentheses) of variables related to the advertising and promotional channels of the quail game farms (n = 21).

Promotion and Advertising Channels	Finishing Farms	Complete-Cycle Farms	Total	*p*-Value
Website	11 (84.6)	7 (87.5)	18 (85.7)	1.000
Participation in livestock and game fairs	8 (61.5)	2 (25.0)	10 (47.6)	0.183
Hunting magazines advertisements	5 (38.5)	2 (25.0)	7 (33.3)	0.656
Livestock related press advertisements	1 (7.7)	0 (0.0)	1 (4.8)	1.000
General press advertisements	1 (7.7)	0 (0.0)	1 (4.8)	1.000
Internet advertisements	3 (23.1)	0 (0.0)	3 (14.3)	0.257

## Data Availability

The data supporting the conclusions of this article will be made available by the authors upon reasonable request.

## References

[1] Puigcerver M., Sanchez-Donoso I., Vilà C., Sardà-Palomera F., García-Galea E., Rodríguez-Teijeiro J.D. (2014). Decreased fitness of restocked hybrid quails prevents fast admixture with wild European quails. Biol. Conserv..

[2] Smith S., Fusani L., Boglarka B., Sanchez-Donoso I., Marasco V. (2018). Lack of introgression of Japanese quail in a captive population of common quail. Eur. J. Wildl. Res..

[3] Puigcerver M., Vinyoles D., Rodríguez-Teijeiro J.D. (2007). Does restocking with Japanese quail or hybrids affect native populations of common quail *Coturnix coturnix*?. Biol. Conserv..

[4] Sánchez García-Abad C., Alonso M.E., Prieto R., González V., Gaudioso V.R. (2009). Una visión sobre la avicultura para la producción de caza en España. Inf. Tec. Econ. Agrar..

[5] Sanchez-Donoso I., Vilà C., Puigcerver M., Butkauskas D., Caballero de la Calle J.R., Morales-Rodríguez P.A., Rodríguez-Teijeiro J.D. (2012). Are farm-reared quails for game restocking really common quails (*Coturnix coturnix*)?: A genetic approach. PLoS ONE.

[6] Sanchez-Donoso I., Huisman J., Echegaray J., Puigcerver M., Rodríguez-Teijeiro J.D., Hailer F., Vilà C. (2014). Detecting slow introgression of invasive alleles in an extensively restocked game bird. Front. Ecol. Evol..

[7] Guyomarc’h J.C., Perennou C., Derégnaucourt S., Tesson J.L., Barbier L., Boutin J.M., Rodríguez-Teijeiro J.D., Puigcerver M., Heredia B., Ranner A. (2009). Common Quail Coturnix coturnix European Union Management Plan 2009–2011.

[8] MITECO (2021). Anuario de Estadística Forestal 2019.

[9] Dalmau A. (1994). Manual de la Codorniz. Cría Industrial y Para la Caza.

[10] Caballero de la Calle J.R., Peña J.C., López Fuentes F., Calle M.I. Estudio de la productividad de la codorniz cinegética *(Coturnix c. coturnix)* criada en cautividad. Proceedings of the XI Jornadas Sobre Producción Animal.

[11] Caballero de la Calle J.R., Peña J.C., Buxadé C. (1997). La explotación cinegética de la codorniz. Producciones Cinegéticas, Apícolas y Otras. Zootecnia Bases de Producción; Animal.

[12] Chazara O., Lumineau S., Minvielle F., Roux D., Feve K., Kayang B., Boutin J.M., Vignal A., Coville J.L., Rognon X. (2006). Étude des risques d’introgression génétique de la caille des blés (*Coturnix coturnix coturnix*) par la caille japonaise (*C. c. japonica*): Comparaison et intégration des données comportementales et moléculaires obtenues dans le sud-est de la France. Les Actes BRG.

[13] Amaral A.J., Silva A.B., Grosso A.R., Chikhi L., Bastos-Silveira C., Dias D. (2007). Detection of hybridization and species identification in domesticated and wild quails using genetic markers. Folia Zool.-Praha.

[14] Riesco G. (2013). Especies Cinegéticas. Instalaciones Para la Cría y Repoblación.

[15] Puigcerver M., Sardà-Palomera F., Rodríguez-Teijeiro J.D. (2012). Determining population trends and conservation status of the common quail (*Coturnix coturnix*) in Western Europe. Anim. Biodivers. Conserv..

[16] Caballero de la Calle J.R., Peña J.C., Calle M.I., Caballero J.V. Análisis diferencial entre el huevo de *Coturnix c. coturnix* y sus híbridos con *Coturnix japonica*. Proceedings of the XII Jornadas Sobre Producción Animal.

[17] Caballero de la Calle J.R., Peña J.C., Calle M.I., Caballero J.V. Análisis diferencial entre sistemas de producción en cautividad de la *Coturnix c. coturnix*. Proceedings of the XIII Jornadas sobre Producción Animal.

[18] González-Redondo P. (2004). Un caso de cambio en el manejo de los recursos cinegéticos: La historia de la cría en cautividad de la perdiz roja en España. Rev. Española Estud. Agrosoc. Pesq..

[19] González-Redondo P., Delgado-Pertíñez M., Toribio S., Ruiz F.A., Mena Y., Caravaca F.P., Castel J.M. (2010). Characterisation and typification of the red-legged partridge (*Alectoris rufa*) game farms in Spain. Span J. Agric. Res..

[20] González-Redondo P., García-Domínguez P. (2012). Typification and characterisation of the pheasant (*Phasianus colchicus*) game farms in Spain. Span J. Agric. Res..

[21] González-Redondo P., Sánchez-Martínez R. (2014). Characterisation of wild rabbit commercial game farms in Spain. World Rabbit. Sci..

[22] Bernardos P. (2018). Personal communication.

[23] Pérez y Pérez F. (1974). Coturnicultura. Tratado de Cría y Explotación Industrial de Codornices.

[24] SPSS Inc (2006). Manual del Usuario de SPSS Base 15.0.

[25] Caballero de la Calle J.R., Buxadé C., Peña J.C. Influencia de la fecha de puesta y el tiempo de almacenamiento sobre la viabilidad del huevo de codorniz cinegética. Proceedings of the VII Jornadas Sobre Producción Animal.

[26] Caballero de la Calle J.R., Peña J.C., Carrión E. Influencia de las características del huevo de la codorniz cinegética sobre la morfología del pollito. Proceedings of the VIII Jornadas Sobre Producción Animal.

[27] Caballero de la Calle J.R., Peña J.C., Calle M.I., Caballero J.V. Análisis de la morfología del pollito de codorniz europea y sus híbridos de codorniz japonesa. Proceedings of the XII Jornadas Sobre Producción Animal.

[28] Toschi A. (1959). La quaglia. Vita—Caccia—Allevamento. Ric. Zool. Appl. Caccia.

[29] Boleli I.C., Morita V.S., Matos J.B., Thimotheo M., Almeida V.R. (2016). Poultry egg incubation: Integrating and optimizing production efficiency. Braz. J. Poult. Sci..

[30] Gorrachategui M., Garcia-Rebollar P., González-Mateos G., de Blas C. (1996). Alimentación de aves alternativas: Codornices, faisanes y perdices. Proceedings of the XII Curso de Especialización FEDNA: Avances en Nutrición y Alimentación Animal.

[31] Pagés A., García E. (1991). Enseñanzas y coloquios sobre la perdiz roja. Sel. Avícolas.

[32] López-Ontiveros A. (1994). Caza, actividad agraria y geografía en España. Doc. d’Anàlisi Geogràfica.

[33] Cepero R. (2009). Avicultura alternativa, ¿retorno al pasado, o un camino al futuro?. Sel. Avícolas.

[34] Blanco P.J., Buxadé C. (1995). Explotación de la codorniz. Avicultura Clásica y Complementaria Zootecnia Bases de la Producción Animal.

[35] Berrama Z., Souames S., Merati R., Korteby H.M., Chirane M.S., Negab N., Hettab K., Idris H., Morzougla N., Temim S. (2021). Effects of laying cycle periods on egg quality, egg chemical composition, and reproductive performance of Japanese quail breeders reared in Northern Algeria. World Vet. J..

[36] Price E.O. (2002). Animal Domestication and Behavior.

[37] Ministerio de la Presidencia, Relaciones con las Cortes y Memoria Democrática (2021). Real Decreto 637/2021, de 27 de julio, por el que se establecen las normas básicas de ordenación de las granjas avícolas. Boletín Of. Estado.

[38] El Sabry M.I., Hassan S.S.A., Zaki M.M., Stino F.K.R. (2022). Stocking density: A clue for improving social behavior, welfare, health indices along with productivity performances of quail (*Coturnix coturnix*)—A review. Trop. Anim. Health Prod..

[39] Gharaoghlan M.F., Bagherzadeh-Kasmani F., Mehri M., Ghazaghi M. (2022). The effect of short, long, natural, and intermittent short photoperiods on meat-type Japanese quails. Int. J. Biometeorol..

[40] Puigcerver M., Sánchez-Donoso I., Vilà C., Sardà-Palomera F., Morales-Rodríguez P.A., Caballero de la Calle J.R., Rodríguez-Teijeiro J.D. (2013). Hibridación entre la codorniz común (*Coturnix coturnix*) y la codorniz de granja: Estado de un problema de conservación. Ecosistemas.

[41] Región de Murcia (2021). Proyecto de Decreto de 2021 por el que se Regula el Procedimiento de Certificación Genética y se Regulan las Sueltas y Repoblaciones de Aves Galliformes Autóctonas (Perdiz Roja y Codorniz Común) en la Región de Murcia. Consejería de Agua, Agricultura, Ganadería, Pesca y Medio Ambiente. Región de Murcia. https://cazaypesca.carm.es/web/cazaypesca/programa-perdiz.

[42] Spanish Government (2022). Proyecto de Ley de protección, derechos y bienestar de los animales. Boletín Of. Cortes Gen. Ser. A.

[43] Arroyo B., Delibes-Mateos M., Caro J., Estrada A., Mougeot F., Díaz-Fernández S., Casas F., Viñuela J. (2013). Efecto de la gestión para las especies de caza menor sobre la fauna no cinegética. Ecosistemas.

[44] Volfová M., Palme R., Machovcová Z., Voslářová E., Lukešová G., Večerek V. (2022). Translocation stress is reflected in corticosterone metabolites in pheasant (*Phasianus colchicus*) droppings. Acta Vet. Brno.

[45] Council of the European Union (2005). Council Regulation (EC) Nº 1/2005 of 22 December 2004, on the protection of animals during transport and related operations and amending Directives 64/432/EEC and 93/119/EC and Regulation (EC) Nº 1255/97. Off. J. Eur. Union.

[46] Brown B.C. (2011). How to Use the Internet to Advertise, Promote and Market Your Business or Website—With Little or No Money.

[47] González-Redondo P., Finzi A. Caracterización del marketing en Internet de las granjas cinegéticas comerciales españolas de conejo de monte. Proceedings of the XXXVIII Symposium de Cunicultura.

